# Predictive Value of R_2_CHA_2_DS_2_-VA Score for 90-Day Functional Outcomes After Endovascular Thrombectomy in Acute Ischemic Stroke

**DOI:** 10.3390/medicina61060998

**Published:** 2025-05-28

**Authors:** Faruk Boyacı, Cetin Kursad Akpınar, Mustafa Kursat Sahin, Murat Akcay, Hasan Dogan, Mustafa Yenercag, Guney Erdogan, Berkant Ozturk, Yankı Boyacı, Orhan Ince

**Affiliations:** 1 Department of Cardiology, Samsun Education and Research Hospital, Samsun University, 33805 Samsun, Turkey; faruk_0601@hotmail.com (F.B.); mustafayenercag@hotmail.com (M.Y.); guneyerdogan8174@hotmail.com (G.E.); berkant52.dr@gmail.com (B.O.); 2 Department of Neurology, Samsun Education and Research Hospital, Samsun University, 33805 Samsun, Turkey; cetin.akpinar@samsun.edu.tr (C.K.A.); hasan.dogan@samsun.edu.tr (H.D.); yankisakin@hotmail.com (Y.B.); 3 Department of Family Medicine, Faculty of Medicine, Ondokuz Mayıs University, 55270 Samsun, Turkey; kursat.sahin@omu.edu.tr; 4 Department of Cardiology, Faculty of Medicine, Ondokuz Mayıs University, 55270 Samsun, Turkey; 5 Department of Cardiology, Istanbul Bagcilar Training and Research Hospital, 34100 Istanbul, Turkey; drorhanince@gmail.com

**Keywords:** stroke, endovascular treatment, CHA_2_DS_2_-VASc score, R_2_CHA_2_DS_2_-VA score, CHA_2_DS_2_-VA score, functional outcomes

## Abstract

*Background and Objectives*: Endovascular treatment (EVT) has been shown to enhance long-term recovery and lower mortality rates in patients with intracranial large vessel occlusion-associated acute ischemic strokes (AISs).We aimed to evaluate the predictive value of the pre-stroke CHA_2_DS_2_-VA, R_2_CHA_2_DS_2_-VA, CHA_2_DS_2_-VASc, and R_2_CHA_2_DS_2_-VASc scores in determining 90-day functional outcomes based on the modified Rankin Scale (mRS). *Methods*: In a single center between 2018 and 2023, 665 AIS patients who underwent EVT and achieved successful reperfusion were screened retrospectively. After inclusion and exclusion criteria, 583 patients were included. Based on 90-day mRS scores, patients were classified into two groups: good (mRS ≤ 2, *n* = 257) and poor functional outcomes (mRS 3–6, *n* = 326). The pre-stroke scores were calculated. *Results*: When ROC curve analysis was performed, R_2_CHA_2_DS_2_-VA demonstrated the highest AUC value (*p* = 0.0443) among these scores. The optimal cutoff score was determined to be 4, yielding a sensitivity of 75.77% and specificity of 93.39%. In multivariable analysis, a higher R_2_CHA_2_DS_2_-VA score was significantly associated with worse outcomes (OR = 1.637, 95%, CI: 2.436–5.510, *p* < 0.001). A longer onset-to-recanalization time (OR = 1.009, 95%, CI: 1.005–1.014, *p* < 0.001) and presence of hyperlipidemia (OR = 2.960, 95%, CI: 1.254–6.988, *p* = 0.01) were correlated with poor prognosis. Higher baseline NIHSS scores were associated with unfavorable outcomes (OR = 1.201, 95%, CI: 1.014–1.422, *p* = 0.034), and this association remained significant for NIHSS scores measured 24 h post-EVT (OR = 1.467, 95%, CI: 1.230–1.748, *p* < 0.001). *Conclusions*: The R_2_CHA_2_DS_2_-VA score demonstrates superior predictive ability for 90-day functional outcomes in AIS patients treated with EVT, surpassing CHA_2_DS_2_-VASc and similar scoring systems.

## 1. Introduction

Acute ischemic stroke (AIS) accounts for the majority of all strokes and remains a major cause of morbidity and mortality among adults worldwide [1]. Approximately 35–40% of AIS cases are caused by intracranial large vessel occlusions (LVOs), which affect substantial portions of brain tissue, resulting in high rates of disability and mortality within the first three months [2,3]. Over the past decade, endovascular treatment (EVT) has become the gold standard for LVO-related AIS due to its ability to restore cerebral perfusion and improve clinical outcomes [4,5]. EVT is most effective when performed within 6 h of symptom onset but has also shown benefits in selected patients treated within 24 h [6,7]. However, despite successful reperfusion, a considerable number of patients fail to achieve favorable functional recovery.

Risk scores designed to identify high-risk patients susceptible to poor outcomes and mortality can aid in the efficient allocation of healthcare resources, optimize hospital stays, and help in planning rehabilitation strategies. The CHA_2_DS_2_-VASc score, along with its modified version incorporating renal function (eGFR) known as R_2_CHA_2_DS_2_-VASc, is widely used to predict thromboembolic risk and guide anticoagulation therapy in patients with atrial fibrillation (AF) [8,9]. High pre-stroke scores have been associated with greater stroke severity and worse clinical outcomes. Several studies have demonstrated that the pre-admission CHA_2_DS_2_-VASc score serves as an independent predictor of major adverse cardiovascular and cerebrovascular events (MACCEs) and all-cause mortality, irrespective of AF status [10,11,12]. However, due to gender-based differences in risk prediction, the latest AF guidelines recommend using CHA_2_DS_2_-VA (excluding the gender criterion) [13]. The components of this score system reflect major cardiovascular risk factors, making them useful beyond AF populations for predicting morbidity and mortality [14]. There are limited data in the literature to evaluate these scores before stroke to predict functional outcomes in acute ischemic stroke patients treated with successful reperfusion. We aim to fill this gap in the literature with our study.

In this study, we aimed to compare the predictive accuracy of pre-stroke CHA_2_DS_2_-VA, R_2_CHA_2_DS_2_-VA, CHA_2_DS_2_-VASc, and R_2_CHA_2_DS_2_-VASc scores for predicting 90-day functional outcomes in AIS patients treated with EVT and successful reperfusion, independent of AF presence.

## 2. Materials and Methods

### 2.1. Study Population

This retrospective observational study analyzed 665 consecutive AIS patients who underwent EVT and achieved successful reperfusion (mTICI ≥ 2b) between the years 2018 and 2023 from our hospital’s stroke center database records. Based on inclusion and exclusion criteria, a total of 583 patients were enrolled in the final analysis.

The inclusion criteria required patients to be aged 18 years or older with large vessel occlusion (LVO) in either the anterior or posterior circulation, confirmed by computed tomographic angiography (CTA) or magnetic resonance angiography (MRA). The time from symptom onset to hospital admission had to be within six hours. Additionally, patients were required to have a pre-stroke modified Rankin Scale (mRS) score of less than 2, a National Institutes of Health Stroke Scale (NIHSS) score of 6 or greater at admission, and an Alberta Stroke Program Early Computed Tomography Score (ASPECTS) of at least 6, based on non-contrast CT or diffusion-weighted MRI. The exclusion criteria included the presence of intracranial hemorrhage or an intracranial tumor at admission. Patients with a history of psychiatric disorders that could interfere with neurological assessment were also excluded. Severe comorbid conditions with an anticipated life expectancy of less than one year were considered grounds for exclusion. Cases with missing mRS follow-up data were not included in the analysis. Additionally, patients who exhibited spontaneous complete reperfusion on initial imaging prior to EVT and those who were transferred to another hospital post-EVT, preventing follow-up, were excluded from this study.

At admission, brain CT scans to patients were routinely obtained to rule out hemorrhagic stroke while MRI with diffusion-weighted imaging was utilized to assess occluded vessels. Intravenous trombolytic therapy was administered to patients who had no contraindications and were admitted within the first 4.5 h after the onset of symptoms. In total, 42% of patients received trombolytic therapy and there was no statistical difference between those who received tPA and those who did not. During the procedure, a 6F long sheath was used. The procedure was conducted either under conscious sedation or general anesthesia, depending on the clinical condition of the patient and the joint decision of the interventionalist and anesthesiologist. The only mechanical thrombectomy techniques used were a stent retriever, the direct aspiration technique, or aspiration with stent retriever techniques. The Solitaire [Covidien, Irvine, CA, USA] or Trevo [Stryker, Fremont, CA, USA] stent retrievers were used for thrombectomy. The thrombectomy technique and stent retriever were selected based on the interventionalist’s preference. In cases of partial or no response, alternative techniques or rescue therapies (e.g., balloon angioplasty, stent implantation, intra-arterial thrombolysis, or intra-catheter tirofiban administration) were considered. Patients with suspected atherosclerotic occlusions underwent balloon angioplasty, with or without stent implantation, as deemed necessary. After EVT, all patients underwent routine CT imaging to rule out intracerebral hemorrhage (ICH).

Patients were categorized into two groups based on their 90-day modified Rankin Scale (mRS) scores: those with good functional outcomes (mRS ≤ 2) and those with poor functional outcomes (mRS 3–6). mRS scores were obtained during outpatient clinic examination in most patients and via telephone interview in a few patients. This study was conducted in accordance with the Helsinki Declaration and was approved by the local ethics committee of our hospital on 8 January 2025 (Approval No: 13 January 2025, date: 8 January 2025).

### 2.2. Clinical, Angiographic, and Laboratory Data Collection

Demographic characteristics, clinical risk factors, laboratory findings, and ischemic stroke-related time intervals (symptom-to-door and symptom-to-recanalization) were extracted from medical records. The patients’ data at first presentation and 90th day follow-up were obtained completely from our hospital’s stroke center database records. Stroke severity was evaluated using the National Institutes of Health Stroke Scale (NIHSS) and the Alberta Stroke Program Early Computed Tomography Score (ASPECTS) for all patients. Stroke causes were categorized according to the TOAST (Trial of Org 10172 in Acute Stroke Treatment) criteria, which classify ischemic stroke subtypes based on clinical presentation and diagnostic findings.

Reperfusion success was evaluated using the Modified Thrombolysis in Cerebral Infarction (mTICI) score, which categorizes the degree of reperfusion as follows: Grade 0 indicates no or minimal reperfusion, while Grade 1 represents partial reperfusion affecting less than 50% of the ischemic territory. Grade 2b is defined as reperfusion of 50% or more of the ischemic territory, whereas Grade 2c refers to near-complete reperfusion, excluding cases with slow flow or minor distal emboli. Grade 3 signifies complete perfusion [15,16,17,18,19,20]. Successful reperfusion was defined as mTICI ≥ 2b.

Renal function was evaluated using the Modified Diet in Renal Disease (MDRD) equation, estimating glomerular filtration rate (GFR) for patients with stable kidney function [21]. Individuals with acute kidney injury were excluded. eGFR was determined as the mean of two separate serum creatinine measurements, taken at emergency admission and on the first day of hospitalization in the neurology department.

Stroke risk factors included heart failure, defined as recent decompensated heart failure regardless of left ventricular ejection fraction. Hypertension was identified by a systolic blood pressure of 140 mmHg or higher, a diastolic blood pressure of 90 mmHg or higher, or prior use of antihypertensive therapy. Diabetes mellitus was diagnosed based on a random glucose level of 200 mg/dL or higher, fasting plasma glucose of at least 126 mg/dL, HbA1c of 6.5% or greater, or pre-admission use of antidiabetic medication. Coronary artery disease was determined by the presence of at least 50% stenosis in a coronary artery or a history of coronary revascularization. Peripheral arterial disease was defined as atherosclerotic disease involving the aorta or non-coronary arteries, with clinical evidence of claudication, diminished pulsations, or prior revascularization. Hyperlipidemia was identified by a total cholesterol level of 240 mg/dL or higher, LDL of at least 130 mg/dL, or triglycerides of 200 mg/dL or more. Smoking history was considered a risk factor if tobacco use occurred within the last six months. Atrial fibrillation was diagnosed through electrocardiography, rhythm strip analysis, ambulatory ECG monitoring, or confirmation by a cardiologist.

### 2.3. Statistical Analysis

Statistical analyses were performed using IBM SPSS Statistics for Windows, Version 25.0 (IBM Corp., Armonk, NY, USA). Categorical parameters were assessed using the Chi-square (χ^2^) test, while continuous parameters were compared using the Mann–Whitney U test. Multivariate logistic regression was employed to identify independent predictors of poor prognosis, with results presented as odds ratios (ORs) and 95% confidence intervals (CIs). Receiver operating characteristic (ROC) analysis was performed to assess the predictive value of the CHA_2_DS_2_-VA, R_2_CHA_2_DS_2_-VA, CHA_2_DS_2_-VASc, and R_2_CHA_2_DS_2_-VASc scores, evaluating their area under the curve (AUC), sensitivity, and specificity. A significance value of *p* < 0.05 was considered statistically important.

## 3. Results

A total of 583 patients were analyzed and categorized into two groups based on their 90-day modified Rankin Scale (mRS) scores. Patients with good clinical outcomes (mRS ≤ 2) accounted for 257 individuals, whereas those with poor clinical outcomes (mRS 3–6) comprised 326 patients. Table 1 provides a detailed summary of demographic, laboratory, and clinical variables. Patients younger than 65 years were significantly more prevalent in the good outcome group compared to the poor outcome group (47.5% vs. 20.2%, *p* < 0.001), whereas those aged 75 years or older were more frequently observed in the poor outcome group (48.5% vs. 16.3%, *p* < 0.001). Additionally, female patients were more commonly found in the poor outcome group compared to the good outcome group (50.6% vs. 42.4%, *p* = 0.049).

The poor outcome group patients had a significantly higher rate of comorbidities, including heart failure (44.2% vs. 13.6%, *p* < 0.001), hypertension (95.1% vs. 73.2%, *p* < 0.001), and diabetes mellitus (51.8% vs. 19.1%, *p* < 0.001). A history of vascular disease was more common in the poor outcome group (60.7% vs. 17.9%, *p* < 0.001); additionally, previous stroke, transient ischemic attack (TIA), or thromboembolism (15.6% vs. 1.6%, *p* < 0.001) were significantly more frequent in the poor outcome group compared to the good outcome group patients.

Patients in the poor outcome group revealed significantly higher blood glucose values compared to the good outcome group, with a median of 169 mg/dL (133–224) versus 124 mg/dL (109–150), (*p* < 0.001). The neutrophil-to-lymphocyte ratio (NLR) was higher in the poor outcome patient group, with value as 7.2 (4.5–11.1) versus 3.3 (2.2–5) (*p* < 0.001). Similarly, C-reactive protein (CRP) values were higher in the poor outcome group compared to the good outcome group, with a median of 13 mg/L (7–22) versus 5.6 mg/L (3.5–11), (*p* < 0.001).

Regarding stroke severity and etiology, both baseline NIHSS scores and NIHSS scores at 24 h post-EVT were significantly higher in the poor outcome group (*p* < 0.001). The baseline NIHSS score was median 18 (16–18) versus 13 (10–16) (*p* < 0.001) and again, 24 h post-EVT NIHSS score was higher in the poor outcome group patients with value as median 12 (10–16) versus 6 (4–8) (*p* < 0.001). Cardioembolic stroke was more frequently associated with poor outcomes, observed in 61% of cases compared to 44.4% in the good outcome group (*p* < 0.001). Conversely, undetermined or other stroke etiologies were more common in the good outcome group, with a prevalence of 28.8% compared to 12.9% in the poor outcome group (*p* < 0.001).

Multivariate logistic regression analysis described several independent predictors of poor clinical outcomes (Table 2). Higher NIHSS scores at baseline were related to an increased risk of poor outcomes (OR = 1.201, *p* = 0.034), and this association remained significant for NIHSS scores measured 24 h post-EVT (OR = 1.467, *p* < 0.001). A longer symptom-to-recanalization time was also a significant predictor (OR = 1.009, *p* < 0.001). Patients with lower mTICI scores exhibited worse outcomes compared to those with complete reperfusion (mTICI 3), with mTICI 2b associated with an odds ratio of 7.748 (*p* = 0.009) and mTICI 2c with an odds ratio of 4.162 (*p* = 0.002). The presence of hyperlipidemia increased the likelihood of poor outcomes (OR = 2.960, *p* = 0.01). Finally, a higher R_2_CHA_2_DS_2_-VA score was strongly related to poor prognosis (OR = 3.664, *p* < 0.001) (Table 2).

**Figure 1:**
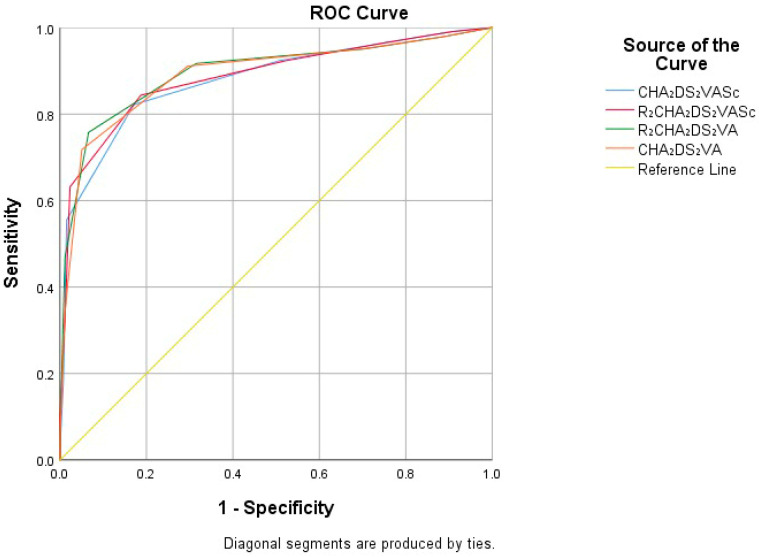
ROC analysis evaluation for CHA_2_DS_2_-VASc, R_2_CHA_2_DS_2_-VASc, R_2_CHA_2_DS_2_-VA, and CHA_2_DS_2_-VA risk scores.

All four risk scores (CHA_2_DS_2_-VA, R_2_CHA_2_DS_2_-VA, CHA_2_DS_2_-VASc, and R_2_CHA_2_DS_2_-VASc) were significantly related to poor prognosis (*p* < 0.001). The predictive value of risk scores was evaluated using receiver operating characteristic (ROC) analysis for CHA_2_DS_2_-VASc, R_2_CHA_2_DS_2_-VASc, R_2_CHA_2_DS_2_-VA, and CHA_2_DS_2_-VA scores. The area under the curve (AUC) value demonstrated their predictive accuracy. The AUC value for these scores were 0.879 for CHA_2_DS_2_-VASc, 0.886 for R_2_CHA_2_DS_2_-VASc, 0.891 for CHA_2_DS_2_-VA, and 0.897 for R_2_CHA_2_DS_2_-VA. The DeLong test confirmed that R_2_CHA_2_DS_2_-VA had the highest AUC value (*p* = 0.0443). The optimal cutoff score for R_2_CHA_2_DS_2_-VA was detected to be 4, yielding a sensitivity of 75.77% and a specificity of 93.39% (Figure 1).

## 4. Discussion

Predicting high-risk patients who are more prone to adverse outcomes and mortality after EVT is crucial for optimizing stroke management. Accurate and easily calculable risk scores would be beneficial in clinical practice for predicting outcomes, prognostic assessment and facilitate decision-making. Our study is the first to compare the CHA2DS2-VASc, R2CHA2DS2-VASc, CHA2DS2-VA, and R2CHA2DS2-VA scores in predicting 90-day functional outcomes assessed by mRS in AIS patients who achieved successful reperfusion after EVT. We demonstrated that the pre-stroke R2CHA2DS2-VA score was superior to other scores in predicting functional outcomes and was significantly and independently associated with these outcomes. Additionally, an R2CHA2DS2-VA score higher than 4 was associated with poor functional outcomes when evaluated using the ROC curve.

With advancements in EVT, successful reperfusion can now be achieved in the majority of cases. Although successful reperfusion strongly predicts a good prognosis in AIS patients, poor functional outcomes remain common. In our study, 55.9% (326/583) of patients who achieved successful reperfusion had poor survival, and 12.5% (73/583) died during the follow-up period. Our findings were consistent with previous studies [15,16]. In multivariate analysis, the R_2_CHA_2_DS_2_-VA score, symptom-to-recanalization time, hyperlipidemia, admission NIHSS score, 24 h post-EVT NIHSS score, and low mTICI score (compared to mTICI 3) were independent predictors of poor functional outcomes. Our findings align with prior research. Studies have shown that patients achieving complete reperfusion (mTICI 3) tend to have better outcomes compared to those with lower mTICI (2b–2c) perfusion [16,17,18,19]. Similarly, studies have shown that a high NIHSS score at admission and 24 h post-EVT, as well as prolonged symptom-to-recanalization time, are associated with poor functional outcomes after AIS [16,17,20,21].

The CHA2DS2-VASc and R2CHA2DS2-VASc scores are widely used, easily repeatable, and effective scoring systems for predicting thromboembolic risk and guiding anticoagulant therapy in non-valvular AF patients [8,9,13]. Some studies in AIS patients have shown that the pre-admission CHA2DS2-VASc score is a strong, independent predictor of MACCEs and all-cause mortality, regardless of AF status [10,11,12]. Two studies in AIS patients undergoing EVT examined the association between the pre-procedural CHA2DS2-VASc score and reperfusion success, assessed by the post-procedural mTICI score. They found that the CHA2DS2-VASc score was significantly higher in patients with failed reperfusion and was independently associated with reperfusion success, regardless of AF presence [22,23]. Recently, Vatan et al.’s [24] study reported that CHA_2_DS_2_-VASc is an independent predictor of futile reperfusion in acute ischemic stroke patients treated with endovascular treatment [24]. Although our study shares similarities with this study, our study population is larger, includes renal function in the scoring, and excludes the impact of female sex on the scoring system.

Age is a significant factor for functional outcomes in acute ischemic stroke. The presence of more comorbidities, impaired collateral circulation, increased microvascular pathology, age-related decreased neurological reserve, and physiological and metabolic changes that hinder recovery may explain worse clinical outcomes in elderly patients after EVT [16,17,25,26,27]. Hypertension (HT) is a major risk and prognostic factor for stroke. HT directly damages the vascular endothelium due to increased shear stress, leading to endothelial remodeling. Endothelial changes impair vasodilation and increase the release of proinflammatory and procoagulant factors. Studies in ischemic stroke patients have demonstrated that HT is a predictor of failed reperfusion and poor functional outcomes [16,17,28]. Diabetes mellitus (DM) increases the risk of ischemic stroke and is related to worse prognosis in terms of mortality and permanent neurological disability compared to non-diabetic patients. Multiple mechanisms link DM to stroke, including vascular endothelial dysfunction, cardiac embolism, atherosclerosis, systemic inflammation, and capillary basement membrane thickening. Studies have shown that DM is associated with poor functional outcomes despite successful reperfusion [16,17,29]. Heart failure (HF) is considered an independent risk factor for ischemic stroke and is also associated with poor clinical outcomes and increased post-stroke mortality. HF contributes to various deleterious pathophysiological mechanisms in AIS, including the release of prothrombotic and proinflammatory mediators and impaired cerebral oxygenation [30].

Chronic kidney disease increases the incidence and severity of stroke. Reduced renal function is related to increased chronic inflammation, anemia, uremia, oxidative stress, and endothelial function. As a result, cerebral autoregulation and collateral circulation are impaired, creating a more vulnerable environment for ischemia. Moreover, renal failure alters hemostatic systems, leading to a prothrombotic state. Studies have shown that renal dysfunction is an independent predictor of functional outcomes in patients treated with EVT [31,32,33].

Despite successful reperfusion after EVT, poor functional outcomes remain a major focus of neurological research. The underlying mechanisms have not been fully elucidated. Possible mechanisms cover lower collateral circulation, high thrombus burden, distal embolization, early reocclusion, inflammatory response, microvascular damage, impaired cerebral autoregulation, and the no-reflow phenomenon [34,35,36,37,38]. Previous studies have demonstrated that the CHA2DS2-VASc score is associated with thrombus burden, increased platelet reactivity, chronic inflammation, microvascular dysfunction, and the slow-flow/no-reflow phenomenon, all of which contribute to worse ischemic stroke recovery. The overlapping pathophysiological pathways of the score components and their cumulative effect lead to increased chronic inflammation and oxidative stress, resulting in endothelial dysfunction and microvascular impairment. Ultimately, impaired cerebral autoregulation and collateral structure create a more vulnerable environment for ischemia, while platelet reactivity and thrombus burden increase.

Among the scoring systems, the R2CHA2DS2-VA score was found to be superior in predicting functional outcomes after EVT. Our study also evaluated the impact of female sex. We found that the predictive ability of the R2CHA2DS2-VA score, which excludes female sex as a factor, was superior. Additionally, the superior predictive ability of R2CHA2DS2-VA compared to CHA2DS2-VA supports the notion that renal function significantly influences functional outcomes after EVT. Multicenter prospective studies are needed for generalizability of findings and their application to real-world daily practice.

Several limitations of this study should be acknowledged. The main limitations include the retrospective design, single-center experience, and small number of patients. In addition, due to the retrospective nature of this study, the possibility of bias regarding the possible effects of treatment/rehabilitation should not be forgotten. This study did not account for pre-stroke antiplatelet and anticoagulant treatments, which could have influenced the results. Many factors influence stroke outcomes, and our study did not consider all possible variables. Stroke outcomes are associated with early rehabilitation, and differences in patients’ access to rehabilitation services could have affected the results. Furthermore, postoperative complications such as aspiration pneumonia and symptomatic intracranial hemorrhage, which may influence prognosis, were not collected or analyzed, which could have affected the results.

## 5. Conclusions

This study established that the R2CHA2DS2-VA score had significant and independent value in predicting 90-day functional outcomes, assessed by mRS, in AIS patients who achieved successful reperfusion after EVT. Moreover, the predictive ability of the R2CHA2DS2-VA score was superior to that of CHA2DS2-VASc, R2CHA2DS2-VASc, and CHA2DS2-VA scores. The R2CHA2DS2-VA score may serve as a simple risk assessment tool, and this finding should be validated with large-scale prospective multicenter studies.

## Figures and Tables

**Table 1:** Demographic, laboratory, and clinical characteristics of the study population.

Variables	Total *n* = 583	Good Outcomes (mRS ≤ 2) *n* = 257	Poor Outcomes (mRS 3–6) *n* = 326	*p* Value *
Age categories				
<65 years, n (%)	188 (32.2)	122 (47.5) ^a^	66 (20.2) ^b^	<0.001
65–74 years, n (%)	195 (33.4)	93 (36.2) ^a^	102 (31.3) ^a^	
≥75 years, n (%)	200 (34.3)	42 (16.3) ^a^	158 (48.5) ^b^	
Sex (female), n (%)	274 (47)	109 (42.4)	165 (50.6)	0.049
Heart failure history, n (%)	179 (30.7)	35 (13.6)	144 (44.2)	<0.001
Hypertension history, n (%)	498 (85.4)	188 (73.2)	310 (95.1)	<0.001
Stroke/TIA/thromboembolism history, n (%)	55 (9.4)	4 (1.6)	51 (15.6)	<0.001
Vascular disease history, n (%)	244 (41.9)	46 (17.9)	198 (60.7)	<0.001
Diabetes history, n (%)	218 (37.4)	49 (19.1)	169 (51.8)	<0.001
CHA_2_DS_2_VA, median (IQR)	3 (2–4)	2 (1–3)	4 (3–5)	<0.001
R_2_CHA_2_DS_2_VA, median (IQR)	3 (2–5)	2 (1–3)	4 (4–5)	<0.001
CHA_2_DS_2_VASc, median (IQR)	4 (2–5)	3 (2–3)	5 (4–5)	<0.001
R_2_CHA_2_DS_2_VASc, median (IQR)	4 (2–5)	3 (2–3)	5 (4–6)	<0.001
Hyperlipidemia, n (%)	293 (50.3)	113 (44)	180 (55.2)	0.007
Atrial fibrillation, n (%)	266 (45.6)	96 (37.4)	170 (52.1)	<0.001
Smoking, n (%)	160 (27.4)	98 (38.1)	62 (19)	<0.001
Glucose (mg/dL), median (IQR)	141 (116–195)	124 (109–150)	169 (133–224)	<0.001
Serum creatinine (mg/dL), median (IQR)	0.9 (0.7–1)	0.8 (0.7–0.9)	0.9 (0.8–1.1)	<0.001
Glomerular filtration rate (μL/dk), median (IQR)	76 (62.3–92)	82.7 (70–96.7)	70.2 (56–84)	<0.001
Hemoglobin (g/dL), median (IQR)	12.7 (11.5–14)	13.2 (12–14.1)	12.4 (11.2–13.8)	<0.001
Neutrophil/lymphocyte ratio, median (IQR)	5 (2.8–8.9)	3.3 (2.2–5)	7.2 (4.5–11.1)	<0.001
CRP (mg/L), median (IQR)	9 (4.5–16)	5.6 (3.5–11)	13 (7–22)	<0.001
NIHSS score at admission, median (IQR)	16 (13–18)	13 (10–16)	18 (16–18)	<0.001
NIHSS at 24 h after EVT, median (IQR)	10 (6–12)	6 (4–8)	12 (10–16)	<0.001
TOAST classification				
Large artery atherosclerosis, n (%)	154(26.4)	69 (26.8) ^a^	85 (26.1) ^a^	<0.001
Cardioembolic, n (%)	313(53.7)	114 (44.4) ^a^	199 (61) ^b^	
Undetermined or others, n (%)	116(19.9)	74 (28.8) ^a^	42 (12.9) ^b^	
Occlusion site				
ICA, n (%)	33(5.7)	19 (7.4) ^a^	14 (4.3) ^a^	<0.001
Carotid—T/L, n (%)	101(17.3)	24 (9.3) ^a^	77 (23.6) ^b^	
Tandem, n (%)	46(7.9)	21 (8.2) ^a^	25 (7.7) ^a^	
MCA-M1, n (%)	282(48.4)	125 (48.6) ^a^	157 (48.2) ^a^	
MCA-M2, n (%)	62 (10.6)	44 (17.1) ^a^	18 (5.5) ^b^	
ACA A1/A2, n (%)	15 (2.6)	6 (2.3) ^a^	9 (2.8) ^a^	
VA/BA/PCA/Combinations, n (%)	44 (7.5)	18 (7) ^a^	26 (8) ^a^	
First pass, n (%)	211 (36.2)	147 (57.2)	64 (19.6)	<0.001
Modified treatment in cerebral infarction (mTICI) score, n (%)				
2B	102 (17.5)	3 (1.2) ^a^	99 (30.4) ^b^	<0.001
2C	201 (34.5)	47 (18.3) ^a^	154 (47.2) ^b^	
3	280 (48)	207 (80.5) ^a^	73 (22.4) ^b^	
Onset-to-door time (minute), median (IQR)	240 (160–300)	180 (140–240)	270 (200–300)	<0.001
Onset-to-recanalization time (minute), median (IQR)	275 (225–340)	245 (200–295)	300 (240–340)	0.007
Mortality, n (%)	73(12.5)	0 (0)	73 (22.4)	<0.001 **

^a,b^: Values in the same row and subtable not sharing the same subscript are significantly different at *p* < 0.05 in the two-sided test of equality for column proportions. * The chi-square test was used for comparing categorical data, while the Mann–Whitney U test was employed for comparing continuous data. ** Fisher exact test was used. NIHSS: National Institutes of Health Stroke Scale; ICA: intracranial internal carotid artery; M1: M1 segment of the middle cerebral artery; TIA: transient ischemic attack; IQR: interquantile range.

**Table 2:** Predictors of poor outcomes in multivariate logistic regression analysis with R_2_CHA_2_DS_2_VA.

Variables	OR (95% CI)	*p* Value
Sex (1)	1.570 (0.535–4.608)	0.412
Hyperlipidemia (1)	2.960 (1.254–6.988)	**0.013**
Atrial fibrillation (1)	1.075 (0.317–3.646)	0.908
Smoking (1)	1.486 (0.469–4.709)	0.501
Glucose	1.003 (0.994–1.011)	0.540
Hemoglobin	0.946 (0.705–1.267)	0.708
Neutrophil/lymphocyte ratio	1.048 (0.945–1.162)	0.377
CRP	1.016 (0.990–1.044)	0.234
NIHSS score at admission	1.201 (1.014–1.422)	**0.034**
NIHSS at 24 h after endovascular thrombectomy (EVT)	1.467 (1.230–1.748)	**<0.001**
TOAST classification Large Artery Atherosclerosis		
Cardioembolic	0.778 (0.190–3.191)	0.727
Undetermined or Others	3.322 (0.754–14.625)	0.112
Occlusion site ICA		
Carotid—T/L	4.528 (0.561–36.514)	0.156
Tandem	1.728 (0.209–14.300)	0.612
MCA-M1	2.119 (0.294–15.253)	0.456
MCA-M2	0.506 (0.051–5.020)	0.560
ACA A1/A2	4.165 (0.211–82.060)	0.348
VA/BA/PCA/Combinations	2.764 (0.160–47.728)	0.484
First pass (no)	1.804 (0.781–4.168)	0.167
Onset to recanalization time	1.009 (1.005–1.014)	**<0.001**
mTICI score 3		
2b	7.748 (1.658–36.204)	**0.009**
2c	4.162 (1.723–10.054)	**0.002**
R_2_CHA_2_DS_2_VA	3.664 (2.436–5.510)	**<0.001**
Constant	0	**<0.001**

Cox and Snell R Square = 0.652; Nagelkerke R Square = 0.874; Accuracy = 94.2.

## Data Availability

The original contributions presented in this study are included in the article; further inquiries can be directed to the corresponding authors.
